# RAI14 silencing suppresses progression of esophageal cancer via the STAT3 pathway

**DOI:** 10.18632/aging.103613

**Published:** 2020-09-14

**Authors:** Jianlin Wang, Yun Cai, Judong Luo, Zhiqiang Sun, Jingping Yu, Feng Yan, Xia He

**Affiliations:** 1 Department of Clinical Laboratory, The Affiliated Cancer Hospital of Nanjing Medical University and Jiangsu Cancer Hospital and Jiangsu Institute of Cancer Research, Nanjing, China; 2 Department of Radiation Oncology, The Affiliated Cancer Hospital of Nanjing Medical University and Jiangsu Cancer Hospital and Jiangsu Institute of Cancer Research, Nanjing, China; 3 Department of Radiotherapy, The Affiliated Changzhou No. 2 People’s Hospital with Nanjing Medical University, Changzhou, China; 4 Department of Pharmacy, The Third Affiliated Hospital of Guangzhou Medical University, Guangzhou, Guangdong, China

**Keywords:** RAI14, esophageal carcinoma, progression, STAT3

## Abstract

Esophageal cancer (EC) is an aggressive malignancy that has an unclear molecular pathogenesis. Although retinoic acid induced 14 (RAI14) is involved in various cancer processes, the relationship between EC and RAI14 has not been elucidated. Our study reported the oncogenic function of RAI14 and its underlying mechanisms in EC. The Cancer Genome Atlas (TCGA) database revealed that RAI14 was upregulated in EC, and this upregulation correlated with T stage, histologic grade, and poor clinical prognosis. RAI14 was evaluated in EC cell lines, and the overexpression of RAI14 promoted cell proliferation, migration, and invasion *in vitro*. Conversely, RAI14 knockdown induced apoptosis and cell cycle arrest. RAI14 activated STAT3, upregulated Mcl-1 and cyclin D1, and inhibited cleaved caspase-3. Inhibition of STAT3 restored the oncogenic effect of RAI14, and RAI14 silencing restrained tumor growth and the protein level of Ki67 *in vivo*. Our results suggest that RAI14 regulates the STAT3 pathway and acts as an oncogene during EC progression.

## INTRODUCTION

Esophageal carcinoma (EC) is one of the most common types of malignant cancers. According to the American Cancer Society, there were 17 650 estimated new cases of EC and 16 080 estimated EC-related deaths in the US in 2019 [1]. The morbidity of EC may be caused by differences in physiological, socioeconomic, or sociodemographic characteristics. Despite the advent of multimodal treatments, including surgery, radiation and chemotherapy, the combined 5-year survival rate of EC at all stages is approximately 19% [2]. Early symptoms of the disease are inconspicuous, and late diagnosis and rapid metastatic spread lead to a higher mortality. Therefore, the investigation of potential targets and molecular mechanisms underlying the development and progression of EC are necessary.

The retinoic acid induced 14 (RAI14) gene, also known as the novel retinal pigment endothelial cell gene (NORPEG), is a developmentally regulated gene that was originally derived from the human retinal pigment epithelial cell line [3]. Investigations have revealed that RAI14 is ubiquitously expressed in human tissues and is involved in a wide range of physiological and pathological processes. Additionally, RAI14 acts as a regulatory protein in the retina, placenta, testes, and spermatozoa [4–6] and also participates in nicotinamide adenine dinucleotide phosphate (NADPH) oxidase-mediated signal transduction in atherosclerosis, the innate immune signaling pathways that regulate type I interferon, the regulation of tight junctions in cells, tumor invasion phenotype, and drug sensitivity [7–9]. Further, recent studies have highlighted the association between RAI14 and malignancies, such as breast cancer [10], gastric cancer [11], and prostate cancer [8]. However, the relationship between RAI14 and EC remains unknown.

Therefore, the current study aimed to evaluate the relationship between RAI14 and EC and the mechanisms underlying this relationship. We demonstrated that RAI14 was upregulated in EC and associated with the malignant behavior of EC. Further, RAI14 silencing suppressed EC progression via the STAT3 signaling pathway; thus, RAI14 may be a valuable prognostic marker and therapeutic target for EC.

## RESULTS

### RAI14 is upregulated in EC and associated with clinicopathological features

**Figure 1:**
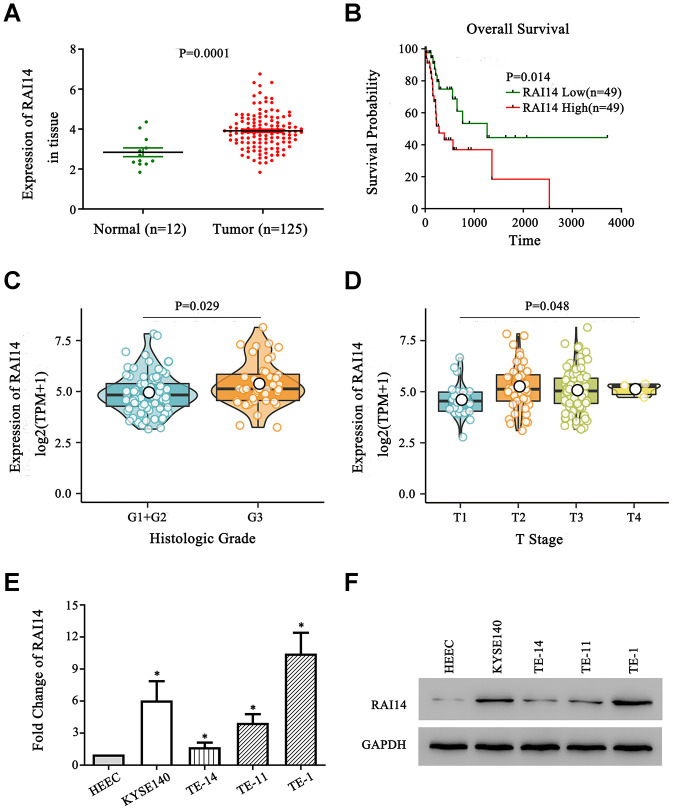
**RAI14 is upregulated in EC.** (**A**) The expression of RAI14 was upregulated in 125 esophageal tumor tissue samples (Tumor) as compared with that in 12 adjacent normal esophageal tissue samples (Normal) in TCGA. (**B**) Kaplan–Meier overall survival curves of EC patients stratified by high and low expression of RAI14. (**C**) The association between the expression of RAI14 and histologic grade. (**D**) The association between the expression of RAI14 and T stage. (**E**) qPCR analysis of RAI14 mRNA levels in EC cell lines and the esophageal epithelial cell line (HECC). (**F**) RAI14 protein levels in the EC cell lines and HECC line were determined by Western blotting. Average RAI14 mRNA and protein levels were normalized to the expression of GAPDH. Three independent experiments were conducted for each assay. *P < 0.05 vs HEEC.

The expression of RAI14 in The Cancer Genome Atlas (TCGA) database was higher in EC tissue than that in paracancerous normal tissue (Figure 1A). Next, a prognostic analysis was conducted using the Kaplan–Meier method, and the overall survival (OS) of patients with low RAI14 expression was increased as compared with that of patients with high RAI14 expression (Figure 1B). Further, the analysis of clinicopathological features showed that RAI14 expression was significantly correlated with T stage and histologic grade (*P* < 0.05) (Figure 1C, 1D). However, RAI14 expression was not correlated with M stage (*P* = 0.24), N stage (*P* = 0.34), and clinical stage (*P* = 0.10) (Data not showed). The expression of RAI14 in the EC cell lines was evaluated by real-time quantitative polymerase chain reaction (qPCR) and western blotting, and the human esophageal epithelial cell (HEEC) line was used as the control. The results from these analyses revealed that levels of RAI14 mRNA and protein were increased in all four EC cell lines as compared with those in the HEEC line (*P* < 0.05) (Figure 1E, 1F). These findings suggest that RAI14 is upregulated in EC and is clinically correlated with a worse prognosis.

### RAI14-modulated cell lines

**Figure 2:**
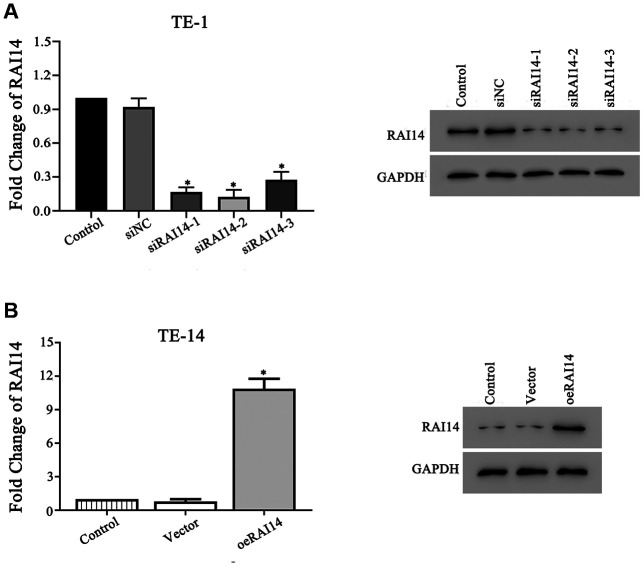
**RAI14 was modulated in EC cell lines.** (**A**) mRNA and protein levels of RAI14 in TE-1 cells transfected with siNC, siRAI14-1, siRAI14-2, or siRAI14-3. (**B**) mRNA and protein levels of RAI14 in TE-14 cells that were transfected with the RAI14-vector or RAI14 sequence. *P < 0.05 vs Control.

The TE-1 cells had the highest expression of RAI14 (10.2-fold) and the TE-14 cells had the lowest expression of RAI14 (1.68-fold) among the selected EC cell lines (Figure 1). To explore the role of RAI14 in EC, we ectopically expressed RAI14 in TE-14 cells and knocked down RAI14 in TE-1 cells. There were no differences in the RAI14 mRNA and protein levels between the cells that were transfected with the control vector or siRNA negative control (siNC) and the control (non-transfected) cells (*P* > 0.05). However, the expression of RAI14 was blocked in TE-1 cells that were transfected with siRNA-1, siRNA-2, or siRNA-3 as compared with that in TE-1 cells that were transfected with siNC (*P* < 0.05) (Figure 2A). Further, the expression of RAI14 was markedly elevated in the RAI14-transfected TE-14 cells as compared with that in the TE-14 vector control cells (Figure 2B). The modulated TE-1 and TE-14 cells were used in subsequent experiments.

### RAI14 expression affects cell proliferation, cell cycle, and apoptosis in EC

The regulatory effects of RAI14 on EC cell proliferation were determined using a Cell Counting Kit-8 (CCK-8) assay, while the Annexin V/PI double-staining assay and PI staining were utilized in apoptotic and cell cycle tests, respectively.

**Figure 3:**
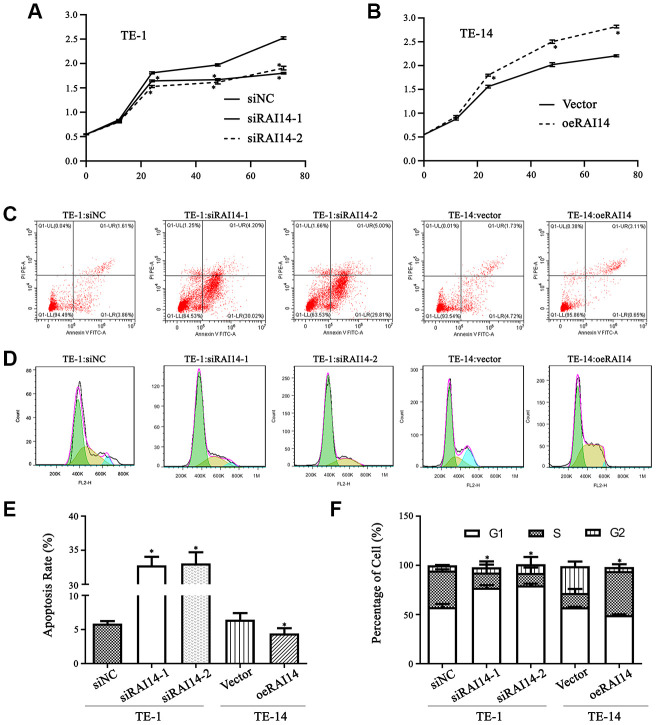
**RAI14 modulates proliferation, apoptosis, and cell cycle in EC.** (**A** and **B**) Cell viability in cells that were transfected with the modulated lentiviruses was detected by the CCK-8 assay. TE-1 cells that were transfected with siNC, siRAI14-1, or siRAI14-2 (**A**); TE-14 cells that were transfected with the RAI14-vector or RAI14 (**B**). *P < 0.05 vs Control group. (**C**) Annexin V–FITC/PI staining discriminated between cells that were in early (lower right quadrant) and advanced (upper right quadrant) apoptotic states. Viable cells were double negative (lower left quadrant). (**D**) Cell cycle distribution after RAI14 modulation was determined. (**E**) The apoptosis rate (including the early and advanced apoptotic states). *P < 0.05: apoptosis rate in the modulation group versus thee respective control. (**F**) Proportion of cells in each cell-cycle phase. *P < 0.05: percentage of cells in S phase in the modulation group versus the respective control.

The proliferation curve of the EC cells shows that there was no notable difference in cell viability between RAI14-knockdown cells and siNC-transfected cells before 12 h. However, at 24, 48, and 72 h after RAI14 knockdown, cell viability was clearly attenuated as compared with that in the siNC-transfected cells (*P* < 0.05). Additionally, there was no significant difference in cell viability between cells that were transfected with siRAI14-1 or siRAI14-2 (Figure 3A). In contrast, cell proliferation in overexpressed-RAI14 cells was enhanced as compared with that in the vector control group (*P* < 0.05) (Figure 3B). These results imply that RAI14 silencing inhibits the proliferation of EC cells.

There was no difference in the apoptosis rate between cells that were transfected with the control vector or siNC and non-transfected cells (*P* > 0.05) (Figure 3C). However, the apoptosis rate in cells that overexpressed RAI14 was markedly reduced as compared with that in the vector-transfected cells (*P* < 0.05), whereas the apoptosis rate in RAI14-knockdown cells was significantly elevated as compared with that in the siNC-transfected cells. These findings suggest that RAI14 silencing induces the apoptosis of EC cells (Figure 3E).

Cells that were transfected with siRNA-RAI14 showed cell cycle arrest at the G1 phase, and the proportion of cells in the S phase that were transfected with siRNA-RAI14 was decreased as compared with in the siNC-transfected cells (*P* < 0.05) (Figure 3D). In the RAI14-transfected group, the proportion of cells in the S phase was significantly increased (44.7 ± 3.2%) as compared with that in the vector-transfected cells (14.5 ± 4.1%) (Figure 3F). These data revealed that overexpressing RAI14 significantly increased the percentage of cells in the S phase, while silencing RAI14 decreased the percentage of cells in S phase, indicating that RAI14 triggered a G1-to-S phase transition in EC.

### RAI14 expression affects cell migration and invasion in EC

**Figure 4:**
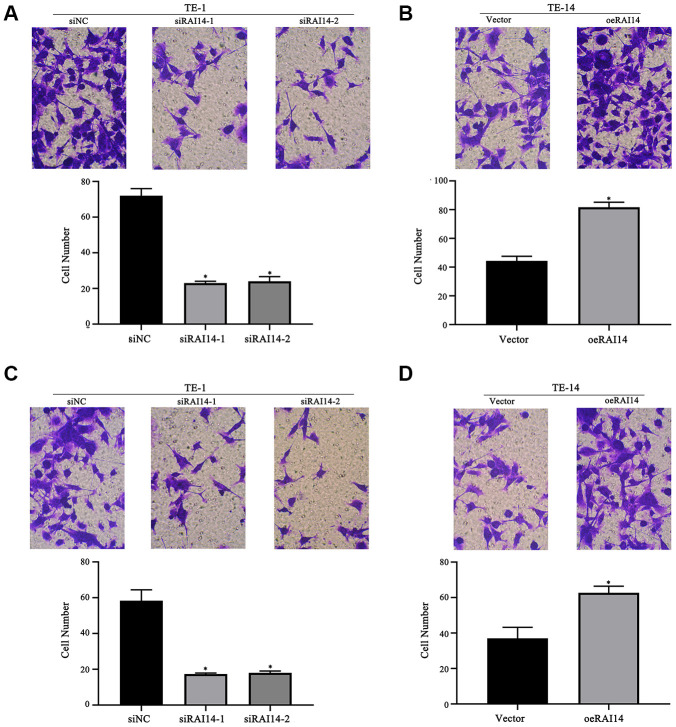
**RAI14 modulates migration and invasion in EC.** (**A**) Migratory ability of TE-1 cells after RAI14 knockdown. (**B**) Migratory ability of TE-14 cells after RAI14 overexpression. (**C**) Invasion ability of TE-1 cells after RAI14 knockdown. (**D**) Invasion ability of TE-14 cells after RAI14 overexpression The results are presented as the mean ± SD of three independent experiments. *P < 0.05 vs Control group.

Results from the transwell assay revealed that the downregulation of RAI14 led to the decreased migration of TE-1 cells as compared with siNC-transfected cells (Figure 4A), and the upregulation of RAI14 increased the migration of TE-14 cells as compared with vector-transfected cells (Figure 4B). Additionally, the downregulation of RAI14 markedly inhibited TE-1 invasion (Figure 4C); however, the invasion ability of the TE-14 cells that were transfected with RAI14 was enhanced (Figure 4D). These results suggest that RAI14 silencing inhibits EC cell migration and invasion.

### RAI14 regulates the STAT3 pathway and its related downstream factors

**Figure 5:**
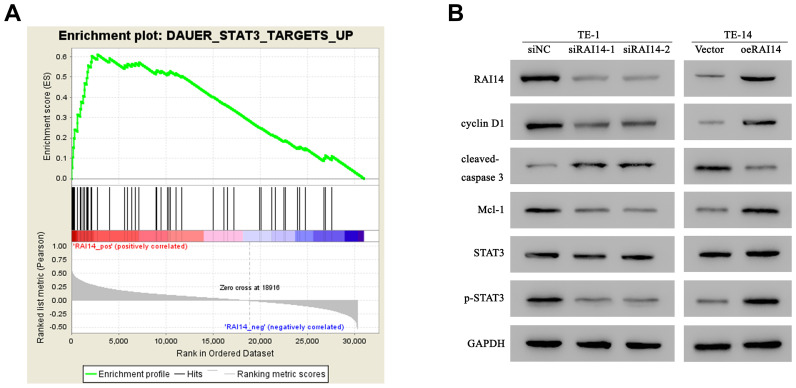
**RAI14 regulates the STAT3 pathway and related factors.** (**A**) The GSEA showed that the level of RAI14 level was positively correlated with STAT3-activated gene signatures (DAUER_STAT3_TARGETS_UP). (**B**) Western blotting of RAI14, p-STAT3, total STAT3, Mcl-1, cyclin D1, and cleaved caspase-3 in RAI14-modulated cells. GAPDH was used as the control.

In order to identify the molecular events that are involved in RAI14 regulation, we investigated its related cell signaling pathways using gene set enrichment analysis (GSEA). Results from the GSEA revealed that the level of RAI14 was positively correlated with the STAT3-activated signatures (Figure 5A). STAT3 is a pivotal cellular pathway that is frequently activated in multiple cancers. Changes in STAT3 activation and factors that are related to cell cycle and apoptosis were detected by western blotting after RAI14 modulation. The ratio of p-STAT3/STAT3 was increased in RAI14-overexpressed cells but decreased in RAI14-silenced cells. Mcl-1 serves as an antiapoptotic factor, while cleaved caspase-3, the activated form of caspase-3, is an important executor in cell apoptosis. In RAI14-deficient-TE-1 cells, the expression of Mcl-1 was reduced and the expression of cleaved caspase-3 was elevated as compared with the expression of Mcl-1 and cleaved caspase-3 in the siNC group (*P* < 0.05). However, the expression of Mcl-1 was increased and cleaved caspase-3 was reduced in TE-14 cells that overexpressed RAI14 as compared with the expression of Mcl-1 and cleaved caspase-3 in the vector control group (*P* < 0.05). Cyclin D1 promotes the transition from G1-to-S and regulates tumorigenesis, and the expression of cyclin D1 was significantly suppressed in RAI14 knockdown-cells as compared with that in the siNC group. Conversely, the expression of cyclin D1 in cells that were transfected with RAI14 was upregulated as compared with that in the vector control group (Figure 5B). These results indicate that the RAI14 regulates STAT3 and its related factors.

### RAI14 promotes the progression of EC by activating STAT3

**Figure 6:**
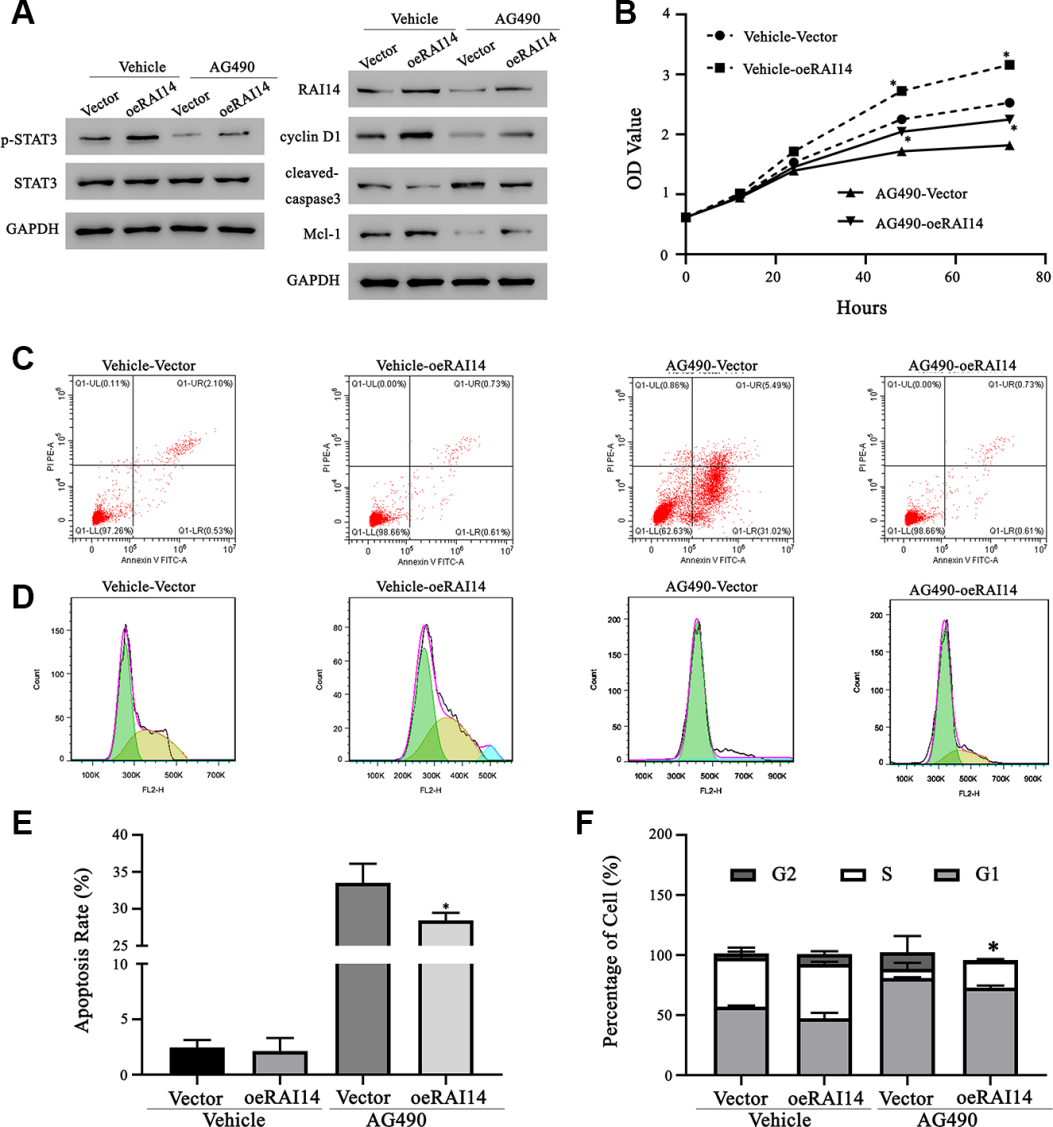
**Regulation of RAI14 in EC proliferation depends on the STAT3 pathway.** The STAT3 inhibitor, AG490, was dissolved in DMSO. TE-14 cells that were transfected with the RAI14-vector or RAI14 were treated with DMSO (Vehicle) or AG490. (**A**) Protein levels of RAI14, p-STAT3, total STAT3, Mcl-1, cyclin D1, and cleaved caspase-3 were determined by western blotting. GAPDH was used as the internal control. (**B**) The proliferation of cells was assessed using the CCK-8 assay. (**C**) Flow cytometry scatter plot of apoptosis. (**D**) Flow chart depicting cell cycle distribution that was assessed by flow cytometry. (**E**) Apoptosis rate. (**F**) Proportion of cells in each cell-cycle phase. *P < 0.05 versus the respective control group.

In the rescue experiment, TE-14 cells that were overexpressed with RAI14 were treated with the STAT3-related inhibitor, AG490, or dimethyl sulfoxide (DMSO; vehicle). The phosphorylation of STAT3 in both the vector-transfected or RAI14-transfected cells that were treated with AG490 was significantly decreased as compared with that in cells that were treated with DMSO. In parallel, the expression levels of Mcl-1 and cyclin D1 were decreased and the level of cleaved caspase-3 was increased in cells that were treated with AG490 as compared with those in cells that were treated with DMSO (Figure 6A). The CCK-8 assay showed that the cell viability in the AG490 treatment group was impeded as compared with that in the vehicle group. However, the proliferation rate of cells that were transfected with RAI14 was higher than that of vector control cells even in the presence of AG490 (Figure 6B). The antiapoptotic effect of RAI14 was rescued in AG490-treated cells (apoptosis rate, 28.4 ± 0.7%) as compared with that in DMSO-treated cells (apoptosis rate, 2.1 ± 0.5%), and AG490 markedly increased the apoptosis rate in the vector control group (at least 13.4-fold) (Figure 6C, 6E). Similarly, results from the cell cycle analysis revealed that AG490 exerted an inhibitory effect on the G1-to-S transition. After incubation with AG490, the proportion of cells in the S phase decreased from 40.7 ± 5.0% to 7.8 ± 5.2% in the vector control group and from 45.2 ± 1.6% to 22.3 ± 1% in the RAI14-transfected group (Figure 6D, 6F). These results suggest that RAI14 modulates tumor proliferation, apoptosis, and cell cycle via the STAT3 pathway.

### RAI14 silencing represses tumor growth *in vivo*

**Figure 7:**
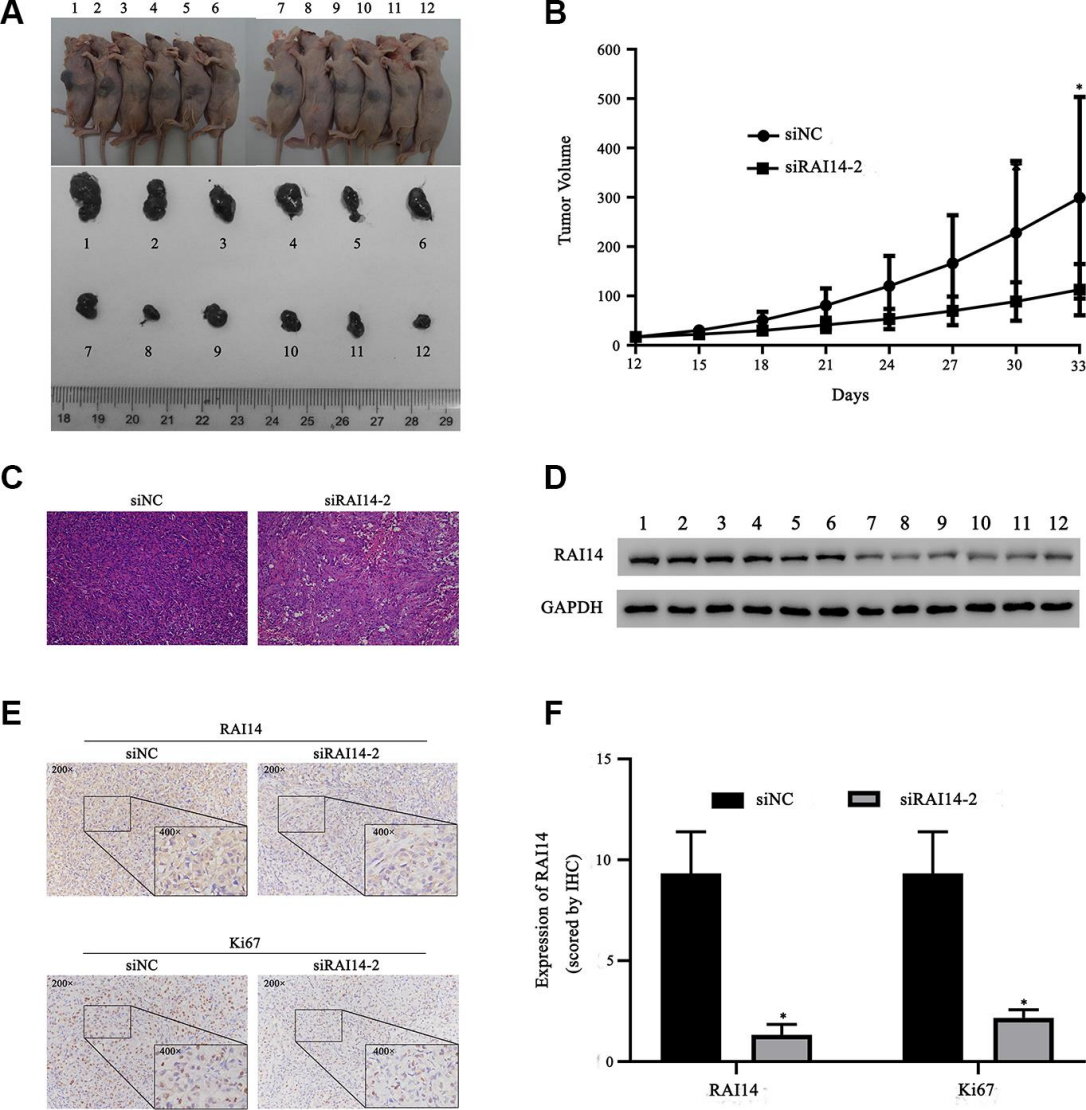
**RAI14 knockdown prevents tumor growth of EC *in vivo.*** Nude mice were injected with TE-1 cells that were transfected with siNC (Nos. 1–6) or siRAI14-2 (Nos. 7–12). (**A**) Tumor volume in mice. (**B**) Growth curve of tumor volume (mm^3^). (**C**) HE staining of tumor samples. (**D**) The protein expression of RAI14 was determined by Western blotting. (**E**) Immunohistochemical results of RAI14 and Ki67 expression in tumor tissue samples. (**F**) The immunohistochemical assay score in each group. *P < 0.05 versus respective control group (siNC).

A xenograft tumor experiment in nude mice was conducted to verify the potential effect of RAI14 on EC. Thirty-three days after inoculation, the tumor volume in the RAI14-silencing group (siRAI14-2) was decreased as compared with that in control group (Figure 7A, 7B). This difference in tumor volume was verified by hematoxylin–eosin (HE) staining (Figure 7C). Western blotting confirmed that the level of RAI14 protein was downregulated in the tumors in the RAI14-knockdown group as compared with that in the control group. Additionally, the cell proliferation rate was lower in the RAI14-knockdown group as compared with that in the control group (Figure 7D). Immunohistochemistry showed that RAI14 protein was mainly localized in the cytoplasm of EC cells (Figure 7E). Ki67 functions as a proliferation marker in cancer, and the levels of RAI14 and Ki67 were significantly decreased in the xenograft tumors in the RAI14-knockdown mice as compared with those in the xenograft tumors in the control mice (Figure 7F) (*P* < 0.05 vs. siNC). These findings suggest that RAI14 silencing suppresses tumor progression *in vivo*.

## DISCUSSION

EC is associated with a high recurrence and mortality [1]; thus, the development of molecular therapeutic targets are critical. RAI14 is ubiquitously expressed in human tissues and is responsible for multiple cell functions [4]. For instance, RAI14 is associated with inflammation in neurons and participates in cytoskeletal functions and drug sensitivity [6, 7, 9]. The utilization of high-throughput sequencing has enabled systematic investigations of genomic alterations in cancer, which have revealed the association between RAI14 and multiple tumor systems [8, 10–12]. However, the relationship between RAI14 and EC has not been completely elucidated.

Results from bioinformatics analysis primarily showed that RAI14 was overexpressed in EC and correlated with a poor prognosis and clinic pathology. Similarly, RAI14 overexpression in breast cancer and gastric cancer were reported to be an independent predictor of poor prognosis [10, 13]. These findings suggest that RAI14 may serve as a clinical prognostic indicator for different cancers.

Here, we found that RAI14 was also upregulated in EC cell lines, and results from gain- and loss-of-function experiments revealed that RAI14 silencing inhibited the proliferation, migration, and invasion of EC cells *in vitro* and suppressed tumorigenesis *in vivo*. Further experiments showed that RAI14 silencing induced apoptosis and cell cycle arrest, indicating the oncogenic function and the multifold mechanical involvement of RAI14 in EC.

The results that are presented in our study are similar to those from previous investigations. For example, Hsu et al. reported that RAI14 was associated with metastatic potential and the sensitivity to chemotherapy [14]. The TMOD3/RAI14/VWF axis interacts with the cytoskeleton in prostate cancer [8], and Liu and colleagues revealed that cell migration and the invasion of melanoma was impaired by RAI14 depletion via regulating the AFAP1-AS1/miR-653-5p/RAI14 axis [15]. Additionally, RAI14 is related to NR2F2 and cell cycle regulation in ovarian cancer [12]; however, inconsistent results were found in lung adenocarcinoma [16]. Specifically, Yuan et al. discovered that RAI14 was overexpressed in lung adenocarcinoma A549 cells and in 31 out of 71 patients, but the cell viability of normal BEAS-2B lung epithelial cells that were transfected with RAI14 was inhibited. This discrepancy was most likely due to the diversity between tumor cells and normal cells. With respect to RAI14, the potential biological mechanisms in EC require the investigation of cell signaling pathways. The GSEA showed that RAI14-mediated EC pathology was correlated with STAT3. The JAK/STAT family is an important component of diverse signal transduction pathways in carcinoma. Among these components, STAT3 is intimately linked to tumorigenesis [17]. In the current study, the phosphorylation of STAT3 was suppressed when RAI14 was silenced in EC cells, and this suppression impeded tumor progression. Additionally, results from the rescue experiment revealed that the inhibition of STAT3 led to an anticancer effect that was similar to the anticancer effect that was observed when RAI14 was silenced. Further, this inhibition even reversed the effect of RAI14 overexpression. To conclude, RAI14 regulates EC progression via the STAT3 pathway.

RAI14 is associated with regulatory molecules that may contribute to the regulation of STAT3. Data have revealed that the amino acid sequences of RAI14 contain nuclear localization and nuclear export signals [4] and that RAI14 is associated with super enhancer [16]. This implies that RAI14 is involved in multiple regulatory functions. Hsu and colleagues [14] revealed that RAI14 was positively correlated with the sensitivity to targeted chemotherapeutic agents, such as dasatinib, which is an inhibitor of Src tyrosine kinase that can activate STAT3 [18]. In addition, the TMOD3/RAI14/VWF axis was verified as a heme oxygenase-1 interacting protein that binds STAT3 [8]. Interleukin (IL)-6 is related to cancer growth and metastasis and activates STAT3 through IL-6Rα and glycoprotein 130. Shen et al. reported that the inhibition of the mammalian target of the rapamycin pathway decreases RAI14 and IL-6 in the U87 glioblastoma cell line, and this suggests that there is a relationship between RAI14 and inflammation [9]. These studies also imply that RAI14 regulates STAT3 by interacting with its upstream factors, including c-Src, heme oxygenase-1, and IL-6.

We further investigated the downstream factors of STAT3 in EC cells. Dysregulated proliferation is a significant hallmark of tumor cells [19], and the regulatory factors include antiapoptotic proteins (e.g., Bcl-xL, and Mcl-1) [20, 21] and proliferation regulatory proteins (e.g., cyclin D1, Myc, and survivin) [22–24].

Cyclin D1 is involved in cell cycle regulation and acts at a limited rate during the G1/S conversion process. Abnormalities in cyclin D1 are common in squamous cell carcinoma and adenocarcinoma [24]. In the current study, the knockdown of RAI14 or inhibition of STAT3 activation reduced the expression of cyclin D1 and ultimately induced cell cycle arrest. This finding is consistent with the results from other studies in which cyclin D1 was downstream of STAT3 [25].

STAT3 localizes to mitochondria and is related to apoptosis [26]. The expression ratio of proapoptotic to antiapoptotic factors in mitochondria is crucial in apoptosis [27]. Specifically, Mcl-1 is an antiapoptotic member of the Bcl-2 family and serves as a sensor of protein synthesis inhibition [26]. p-STAT3 is reported to bind to a serum inducible element (SIE)-like binding site in the mouse Mcl-1 promoter and potentiate Mcl-1 expression in v-src-transformed NIH3T3 cells [20]. Most of the factors that trigger apoptosis ultimately require the participation of caspase-3 [28]. Here, we showed that RAI14 overexpression increased the expression of Mcl-1 but reduced the expression of cleaved caspase-3. Additionally, the depletion of RAI14 or inhibition of the STAT3 pathway induced apoptosis in EC cells. These findings were supported by tumor growth inhibition and Ki67 depletion in tumor issues *in vivo*. Consistently, Leu and colleagues reported that IL-6 protected EC cells from apoptosis by inducing Mcl-1 without affecting Bcl-2, Bcl-xL, and Bax [29]. Further, the IL-6/STAT3/Mcl-1 axis mediates apoptotic resistance in transformed Barrett’s esophageal cells [30].

In summary, our results suggest that RAI14 is a tumor promoter of EC. RAI14 silencing suppresses proliferation, invasion, and migration by inhibiting STAT3 activation and the expression of downstream factors. This study demonstrates that RAI14 plays an important regulatory role in tumorigenesis and may be a new therapeutic target for EC.

## MATERIALS AND METHODS

### Ethics statement

This study was conducted in accordance with the ethical standards, the tenets of the Declaration of Helsinki, and national and international guidelines. The animal study was carried out in strict accordance with the recommendations outlined in the Guide for the Care and Use of Laboratory Animals of the National Institutes of Health. All of the procedures for cell and animal experiments and the detection of tissue sections were approved by the Ethics Committee of Jiangsu Cancer Hospital.

### Bioinformatics prediction

The Cancer Genome Atlas (TCGA; https://cancergenome.nih.gov/) was used to study the expression of RAI14 in EC and normal esophageal tissues. The TCGA identified 125 EC cases and 12 normal esophageal cases that contained information regarding the expression of RAI14. The expression of RAI14 was analyzed using the R language package, and the analysis of RAI14 and clinical pathology was obtained from the TCGA. The overall survival analysis of the abstracted patient data (n=98) was performed using a log-rank test and presented as a Kaplan–Meier survival curve.

The GSEA was performed to identify the biological pathways that are involved in EC pathogenesis. The canonical pathways gene sets (c2.cp.kegg.v5.1.) that were obtained from the Molecular Signatures Database-MsigDB (http://software.broadinstitute.org/gsea/msigdb/collections.jsp#H) were used for enrichment analysis.

### Cell culture

We obtained the HEEC line and the following human esophageal cancer cell lines from the Cell Bank of the Chinese Academy of Sciences (Shanghai, China): KYSE140, TE-1, TE-11, and TE-14 cell line. The cells were cultured in Dulbecco’s modified Eagle’s medium (Gibco, Carlsbad, CA, USA) supplemented with 10% fetal bovine serum (Gibco), 100 U/mL penicillin, and 100 mg/mL streptomycin (Hyclone; Logan, UT, USA) and incubated at 37 °C with 5% CO_2_. The RAI14-modulated TE-14 cells were treated with 0.1 mM AG490 (Selleckchem, Houston, TX, USA) for 48 h for STAT3 inhibition.

### Cell transfection

Cells were transfected with a lentiviruses to knockdown or upregulate RAI14. Briefly, the PLKO.1 plasmid was obtained from Addgene (Watertown, MA, USA), and the cells were transfected with siRNA-RAI14 (siRAI14) or siRNA negative control (siNC). Three human siRNA sequences (siRAI14-1, GCTGCTTCTTGCTGTACAA; siRAI14-2, GATCAGTTCTATACGAGAA; and siRAI14-3, GGAGTTACAAGATAAATTA) were synthesized by Genewiz Inc. (South Plainfield, NJ, USA). For RAI14 overexpression, the lentiviral vector (pLVX-Puro, oeRAI14) and control vector (vector) were obtained from Clontech (Palo Alto, CA, USA).

TE-1 and TE-14 cells were transfected with either of the lentiviral vectors that encoded the sh-RAI14 or RAI14 overexpressed sequences and the corresponding NC vectors. Lipofectamine 2000 Transfection Reagent (Invitrogen, Carlsbad, CA, USA) was used for transfection. The knockdown and overexpression of RAI14 were evaluated by qPCR and western blotting.

### qPCR

Total RNA was extracted using TRIzol Reagent (Invitrogen) and reverse transcribed to cDNA using the RevertAid First Strand cDNA Synthesis Kit (Fermentas, Thermo Fisher, Carlsbad, CA, USA). mRNA expression was detected using SYBR Master Mix (Takara, Dalian, China), and the primers were as follows: RAI14 forward: 5'-CCAGTGCCACCAAACACG-3', reverse: 5'-TTCGGCTGGGCATTTAGAC-3'; GAPDH forward: 5'-AATCCCATCACCATCTTC-3', reverse: 5'-AGGCTGTTGTCATACTTC-3'. Gene expression was examined using qPCR with the ABI 7500 Fast Real-Time PCR System (Applied Biosystems; Carlsbad, CA, USA). The relative expression of the target genes were determined using the comparative Ct method.

### Western blotting

Western blotting was carried out according to the protocol. Briefly, RIPA Lysis Buffer (CWBIO, Shanghai, China) was used to lyse cells and extract protein. The protein concentration was determined using the BCA Protein Assay Kit (Beyotime, Jiangsu, China). Next, 20 μg of protein from each sample was separated by 10% sodium dodecyl sulfate-polyacrylamide gels (SDS-PAGE), transferred to polyvinylidene fluoride membranes, and incubated with the following primary antibodies overnight: anti-RAI14, anti-Mcl-1, anti-cleaved caspase-3, anti-cyclin D1, anti-STAT3, anti-p-STAT3 and anti-GAPDH. The following day, the membranes were incubated with the following secondary antibody for 1 h: Goat Anti-Rabbit IgG H&L (HRP) (Abcam, Cambridge, MA, USA). An enhanced chemiluminescence kit (Beyotime) was used for signal development, and densitometry was calculated using Image J software. The ratio of the investigated protein to GAPDH was used to reflect the relative expression of the protein.

### Cell proliferation assay

Cell proliferation was determined using a CCK-8 assay. Approximately 10^3^ cells/well were seeded into 96-well plates and incubated for 12, 24, 48, and 72 h. Then, 10 μL of the CCK-8 reagent (SABiosciences, Qiagen, CA, USA) was added to each well, and the cells were incubated for 90 min at 37 °C. The absorbance was detected at 450 nm using the Infinite 200 Pro microplate reader (Tecan; Männedorf, Switzerland).

### Cell apoptosis assay

Flow cytometry was carried out for the detection of apoptosis using the Annexin V-FITC-PI Apoptosis Detection Kit (Beyotime). Briefly, the cells were resuspended in 1× binding buffer at a concentration of 1×10^6^–5×10^6^ cells/mL. Cell suspensions (100 μL) were incubated with 5 μL of Annexin V–fluorescein isothiocyanate (FITC) for 5 min in the dark followed by 10 μL of propidium iodide (PI) and 400 μL of PBS. The samples were analyzed using the FACSCalibur instrument and BD FACSDiva software (BD Bioscience, San Diego, CA, USA).

### Cell cycle analysis

Cells were fixed with 70% ethanol at 4 °C for 12–16 h and stained with PI (Thermo Fisher, Carlsbad, CA, USA). The percentage of cells in different cell cycle phases was analyzed using a Flow Cytometry System (BD Bioscience, Bedford, MA, USA).

### Transwell assay

The transwell assay was used to evaluate cell migration and invasion. To determine cell migration, 5× 10^4^ cells were seeded into the upper chamber in serum-free culture medium (200 μl), and the lower chamber was filled with complete medium. The cells were fixed with 4% paraformaldehyde and then stained with Giemsa 15 min, after 24 h incubation. Cell images were acquired under a microscope, and the migrated cells were counted in 5 random fields. Cell invasion was determined using a method that was similar to the method that used for cell migration; however, the upper chamber was coated with Matrigel (BD Bioscience, Bedford, MA, USA).

### *In vivo* experiments

Four- to five-week-old female BALB/c athymic nude mice (15–17 g) were purchased from Beijing HFK (Bioscience Co. Ltd.) and bred in specific-pathogen-free conditions. After the nude mice adapted to the environment for 1 week, 5×10^6^ TE-1 cells that were in the exponential growth phase were resuspended in 200 μL of PBS and subcutaneously injected into each mouse. Twelve xenograft mice were randomly divided into two groups: the siNC group was injected with TE-1 cells that were transfected with the control vector, and the siRAI14 group was injected with TE-1 cells that contained the siRAI14-2 sequence. Subsequently, tumor volumes were analyzed every 3 d using an electronic caliper and quantitated using the following formula: 0.52 × length × (width)^2^. After treatment for 33 d, the mice were killed, solid tumors were collected, and all samples were analyzed by western blotting and hematoxylin–eosin (HE) staining. All efforts were made to minimize animal suffering.

### Immunohistochemistry

Immunohistochemical staining was performed on paraffin-embedded tissues according to the manufacturer's instructions (EnVision kit, Changdao Biotechnology Co., Itd, Shanghai, China). The following primary antibody was used: rabbit anti-human RAI14 monoclonal antibody (1:150, Abcam, Cambridge, UK). The immunohistochemical scoring principle was based on staining intensity (no signal = 0, weak = 1, moderate = 2, high = 3) and the percentage of stained cells (0%-5% = 0, 6%-25% = 1, 26%-50% = 2, 51%-75% = 3, 76%-100% = 4). The final scores ranged from 0-12 and were calculated by multiplying the intensity scores by the percentage scores. RAI14 staining scores that were ≥ 4 were classified as high expression, and RAI14 staining scores that were < 4 were classified as low expression.

### Statistical analysis

The data are representative of three independent experiments and expressed as the mean ± SD. Survival curves were plotted using the Kaplan-Meier method and compared using the log-rank test. The associations between RAI14 expression and the clinicopathological characteristics of the patients were analyzed using the t-test. A one-way analysis of variance was used to compare differences between three or more groups, and multiple comparisons were conducted using the Student–Newman–Keuls post-hoc test when applicable. Comparisons between the two groups were analyzed using a Student’s t-test. SPSS 22.0 software was used to analyze the data, and *P* < 0.05 was considered statistically significant.
